# Exploring Molecular Equilibrium Geometries in Static and Quantized Fields

**DOI:** 10.1021/acs.jctc.5c00739

**Published:** 2025-10-07

**Authors:** Marcus T. Lexander, Jan Haakon M. Trabski, Andrea Bianchi, Eirik F. Kjønstad, Tor S. Haugland, Henrik Koch

**Affiliations:** † Department of Chemistry, 8018 Norwegian University of Science and Technology, NTNU, Trondheim 7491, Norway; ‡ Scuola Normale Superiore, Piazza Dei Cavalieri 7, Pisa 56126, Italy

## Abstract

Experimental studies have shown that external electromagnetic fields can offer a means to modify molecular structure and reactivity. In this work, we derive and implement analytical molecular gradients at the Hartree–Fock level for both static and quantized electromagnetic fields to explore the field effects on equilibrium geometries. We investigate the equilibrium geometries and field-induced orientations of representative molecules: water, the water dimer, and corannulene. Our analysis reveals distinct field-induced effects: shifts in the equilibrium geometry of water under a combined cavity and static electric field; a reduction in the inversion barrier of corannulene in the presence of quantized or magnetic fields; and orthogonal orientation of the individual water molecules in the water dimer with respect to the quantized field component.

## Graphical abstract

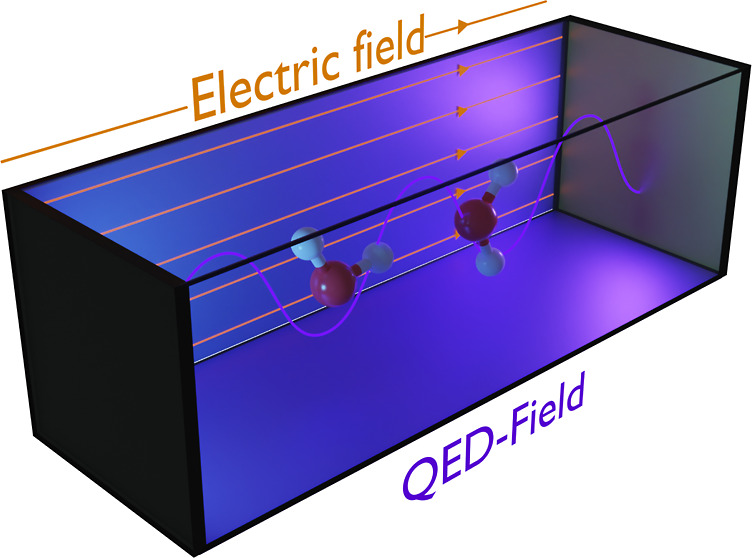

## 1 Introduction

External electromagnetic fields can significantly modify chemical reactions, offering a means to manipulate molecular structures and control reaction pathways.^1–6^ External fields exert a dual effect: they introduce a pronounced orientation dependence, while also influencing internal degrees of freedom. Understanding these effects is highly valuable for predicting changes in molecular properties.^7–9^

Recently, Zhu et al.^10^ demonstrated that halogen bonding complexes give a significant improvement in reaction rates and selectivity when applying a strong electric field (typically around 1.4 V/nm). Theoretical calculations support these findings and explain that the orientation of the molecules plays a key role in lowering the barrier to heterolytic cleavage.^1^ In another study, Cassone et al.^11^ presented computational studies on the use of strong static electric fields to enable the production of methane and formaldehyde, which could serve as an important part of producing green combustion fuels. More recently, Cassone and Martelli.^12^ presented studies on phase changes to bulk water at room temperature, known as electrofreezing.

External magnetic fields have also been shown to alter the electronic structure of molecules. Recently, Wang et al.^2^ investigated CO_2_ reduction to formic acid catalyzed on Cu_2_O nanocubes subjected to a magnetic environment (1 T). A significant improvement in catalytic activity was observed and a separate study supports these findings.^13^ For extreme magnetic fields (1000 T) surrounding neutron stars, theoretical simulations explore the chemical changes in molecules and provide interpretation of observed molecular spectra.^14^ Cavity quantum electrodynamics (QED) has gained increasing attention, because of the ability to create polaritons when molecules interact strongly with a quantized field.^15,16^ In particular, Fabry-Pérot cavities have been used in experimental studies of modifications in selectivity and reaction rates.^3,4^ Various groups proposed that by leveraging two-directional fields, reactivity and selectivity can be tuned simultaneously.^17–19^ They argue that aligning an electric field along the reaction-axis, the direction in which electrons reorganize during a reaction can enhance catalysis, while orienting it along a specific bond axis can induce bond dissociation or shortening. Building on this idea, a possibility is to integrate external electric fields with quantum electrodynamics (QED) by applying a voltage across cavity mirrors. Typical electric field studies use strengths of 0.1–10 V/nm,^10^ while optical cavities range from 100 to 1200 nm, depending on whether electronic or vibrational strong coupling is targeted.^3^

Theoretical studies are essential to understand the effects of external fields on molecular systems. Accurately determining equilibrium molecular geometries is a fundamental prerequisite for any theoretical study. External fields can significantly alter equilibrium geometries, consequently affecting reaction enthalpies and barrier heights, as demonstrated computationally by Pavošević et al.^8^ Furthermore, Liebenthal and DePrince.^7^ highlighted that geometry relaxation is essential for reliably obtaining equilibrium structures in the presence of fields. To determine equilibrium geometries, it is crucial to develop reliable methods for evaluating analytical gradients in the presence of external fields. Analytical electric field molecular gradients can be smoothly evaluated with minimal modifications to the theory.^20^ However, in magnetic fields, the situation is more complex, as obtaining origin-invariant energies and gradients requires the use of London atomic orbitals (LAOs).^21,22^

Similarly, the implementation of analytical gradients in quantum electrodynamics (QED) environments can be complicated, depending on the level of theory. Analytical QED molecular gradients have already been developed for several theoretical models, including the cavity Born–Oppenheimer approximation Hartree–Fock (CBOA-HF),^23^ quantum electrodynamical density functional theory (QEDFT),^7^ and quantum electrodynamical coupled cluster (QED-CCSD) theory.^24^ In this work, we present the analytical derivation and implementation of the quantum electrodynamics Hartree–Fock (QED-HF) molecular gradient. The QED-HF method is equivalent to CBOA-HF with a minimized displacement field.^25^

Beyond the scope of molecular gradients, the coordinate system should be adapted for calculations in external fields. A straightforward approach is to use Cartesian coordinates. However, this may introduce numerical instabilities and convergence issues.^26–28^ Adopting an internal coordinate system can reduce the number of geometry optimization cycles and enhance numerical stability. However, to account for the orientational dependence of the external field, it is essential to include rotational degrees of freedom. The translational-rotational-internal coordinate (TRIC) system developed by Wang and Song.^26^ effectively addresses this problem, providing a more stable and efficient optimization framework, and has been used in the above-mentioned works, and interfaces have been implemented for PSI4,^29^ NWChem^30^ and eT.^31^

This paper is structured as follows: Sections 2 and 3 present a detailed derivation of the QED-HF molecular gradient, while Section 4 extends the theory to include external fields. In Section 5, we present the results for water, corannulene, and water dimer. Finally, in Section 6, we provide our concluding remarks.

## 2 QED-HF Theory

The Pauli-Fierz Hamiltonian in the coherent state basis is expressed as

1 $$\begin{array}{ll} H=H_{e} & +\sum_{\alpha }(\omega_{\alpha }b_{\alpha }^{†}b_{\alpha }+\frac{1}{2}{\left[\lambda_{\alpha }\cdot \left(d-\left\langle d\right\rangle \right)\right]}^{2}\\ & -\sqrt{\frac{\omega_{\alpha }}{2}}\left[\lambda_{\alpha }\cdot \left(d-\left\langle d\right\rangle \right)\right)\left(b_{\alpha }^{†}+b_{\alpha }\right)] \end{array}$$

where the *H*_*e*_ is the Born–Oppenheimer electronic Hamiltonian expressed in second quantization

2 $$H_{e}=\sum_{pq}h_{pq}E_{pq}+\frac{1}{2}\sum_{pqrs}g_{pqrs}e_{pqrs}+h_{nuc}$$

we use the indices *p*, *q*, *r*, and *s* to refer to the molecular orbitals and the additional contributions are summed over the cavity modes α. The first term inside the summation is the pure photonic energy contribution, with the bosonic creation (*b* ^†^) and annihilation (*b*) operators, and the frequency ω_α_ of mode α, and **λ**_α_ is the coupling strength vector. The second term is the dipole self-energy term, and the last term is the bilinear term, which correlates electrons and photons. The dipole operator is defined as

3 $$d=\sum_{pq}d_{pq}E_{pq},,d_{pq}=\langle p|d_{e}+\frac{d_{nuc}}{N_{e}}|q\rangle$$

that is composed of the electronic ***d***_*e*_, the constant nuclear contribution ***d***_nuc_ and where *N*_*e*_ is the number of electrons. The QED-HF reference state is written as

4 |R⟩=|HF⟩⊗|0⟩

where |0⟩ is the photon vacuum state. With this reference state the bilinear term of eq 1 disappears in the expectation value, leaving only the dipole self-energy as an additional contribution to the energy

5 $$E_{QED‐HF}=E_{HF}+\frac{1}{2}\sum_{\alpha }\langle HF|\lambda_{\alpha }\cdot {\left(d-\left\langle d\right\rangle \right)}^{2}|HF\rangle$$

Further details can be found in Haugland et al.^15^ The dipole self-energy term in eq 1 can be written as

6 $$\begin{array}{l} \frac{1}{2}{\left(\lambda_{\alpha }\cdot \left(d-\left\langle d\right\rangle \right)\right)}^{2}=\frac{1}{2}{\left(\lambda_{\alpha }\cdot d\right)}^{2}-\left(\lambda_{\alpha }\cdot \left\langle d\right\rangle \right)\left(\lambda_{\alpha }\cdot d\right)+\frac{1}{2}{\left(\lambda_{\alpha }\cdot \left\langle d\right\rangle \right)}^{2}\\ ,=\sum_{pq}\left(\frac{1}{2}\sum_{r}\left(\lambda_{\alpha }\cdot d_{\mathrm{pr}}\right)\left(\lambda_{\alpha }\cdot d_{rq}\right)-\left(\lambda_{\alpha }\cdot \left\langle d\right\rangle \right)\left(\lambda_{\alpha }\cdot d\right)\right)E_{pq}\\ ,+\frac{1}{2}\sum_{pqrs}\left(\lambda_{\alpha }\cdot d_{pq}\right)\left(\lambda_{\alpha }\cdot d_{rs}\right)e_{pqrs}+\frac{1}{2}{\left(\lambda_{\alpha }\cdot \left\langle d\right\rangle \right)}^{2} \end{array}$$

where we have used the relation

7 $$E_{pq}E_{rs}=e_{pqrs}+\delta_{qr}E_{ps}$$

to expand the square of the dipole operator. This expression is convenient because it allows us to combine the dipole integrals with the one- and two-electron integrals in the electronic Hamiltonian. This subsequently leads to the modified one- and two-electron integrals.

8 $$h_{pq}=h_{pq}^{e}+\sum_{\alpha }\left(\frac{1}{2}\sum_{r}\left(\lambda_{\alpha }\cdot d_{\mathrm{pr}}\right)\left(\lambda_{\alpha }\cdot d_{rq}\right)-\left(\lambda_{\alpha }\cdot \left\langle d\right\rangle \right)\left(\lambda_{\alpha }\cdot d\right)+\frac{\delta_{pq}}{2N_{e}}{\left(\lambda_{\alpha }\cdot \left\langle d\right\rangle \right)}^{2}\right)$$

9 $$g_{pqrs}=g_{pqrs}^{e}+\sum_{\alpha }\left(\lambda_{\alpha }\cdot d_{pq}\right)\left(\lambda_{\alpha }\cdot d_{rs}\right)$$

The QED-HF energy can be expressed in terms of these modified integrals using the same expression as for electronic Hartee−Fock

10 $$E_{QED‐HF}=2\sum_{i}h_{ii}+\sum_{ij}\left(2g_{iijj}-g_{ijji}\right)+h_{nuc}$$

where the indices *i*, *j*, *k*, *l* labels occupied molecular orbitals. This is convenient because it simplifies the derivation and implementation of the QED-HF molecular gradient, as we can reuse the molecular gradients for electronic Hartree–Fock together with some additional terms.

## 3 The QED-HF Gradient

We derive the QED-HF gradient expression in the atomic orbital (AO) basis. The energy expression in eq 10 is transformed to the AO-basis and we obtain the following expression similar to standard Hartree–Fock.^32^

11 $$E_{QED‐HF}=Tr\left(2Dh+DG\left(D\right)\right)+h_{nuc}$$

here ***D*** is the AO density matrix given by

12 $$D_{\mu \nu }=\sum_{i}C_{\mu i}C_{\nu i}$$

where the ***C*** contains the molecular orbital (MO) coefficients and μ and ν are AO-indices.^33^ The one-electron integral matrix ***h***, is the QED-modified integrals in eq 8, and ***G*** (***D***) is the QED modified two-electron integrals contracted with the density

13 $$G{\left(D\right)}_{\mu \nu }=\sum_{\rho \sigma }D_{\rho \sigma }\left(2g_{\mu \nu \rho \sigma }-g_{\mu \sigma \rho \nu }\right)$$

here we use a shorthand notation for the dipole matrix for a given mode *d*_α_

14 $${\left(d_{\alpha }\right)}_{\mu \nu }=\lambda_{\alpha }\cdot d_{\mu \nu }$$

with

15 $$g_{\mu \nu \rho \sigma }=g_{\mu \nu \rho \sigma }^{e}+\sum_{\alpha }{\left(d_{\alpha }\right)}_{\mu \nu }{\left(d_{\alpha }\right)}_{\rho \sigma }$$

The one-electron integrals in eq 8 can be written as in the AO-basis as

16 $$h=h^{e}+\sum_{\alpha }\left(\frac{1}{2}d_{\alpha }S^{-1}d_{\alpha }-\left\langle d_{\alpha }\right\rangle d_{\alpha }+\frac{1}{2N_{e}}{\left\langle d_{\alpha }\right\rangle }^{2}S\right)$$

where ***S*** is the AO-overlap matrix and the *S* ^–1^ arises from the identity

17 $$S_{\alpha \beta }^{-1}=\sum_{s}C_{\alpha s}C_{\beta s}$$

The expression of the molecular gradient is similar to the standard expression^34^

18 $$E^{\left(1\right)}=Tr\left[2Dh^{\left(1\right)}+DG^{\left(1\right)}\left(D\right)-2DFDS^{\left(1\right)}\right]+h_{nuc}^{\left(1\right)}$$

where the superscript (1) denotes the derivative with respect to the nuclear coordinates. The notation ***G*** ^(1)^(***D***) denotes that only contributions from the derivatives of two-electron integrals are included and does not include the derivatives of the density. The third term arises from the orbital connection, keeping the MOs orthonormal. The Fock matrix ***F*** is given by the expression^32^

19 F=h+G(D)

In standard Hartree–Fock the expression in eq 18 is implementable as it only depends on the derivative of the AO integrals. However, in QED-HF, the ***h*** and ***G*** contain the dipole contributions, and we cannot directly differentiate them. To calculate these contributions, we begin by expanding the derivative of the one-electron integrals

20 $$\begin{array}{l} Tr\left(2Dh^{\left(1\right)}\right)=Tr\left[2D{\left(h^{e}\right)}^{\left(1\right)}\right]\\ ,+\sum_{\alpha }Tr[Dd_{\alpha }^{\left(1\right)}S^{-1}d_{\alpha }+Dd_{\alpha }{\left(S^{-1}\right)}^{\left(1\right)}d_{\alpha }+Dd_{\alpha }S^{-1}d_{\alpha }^{\left(1\right)}\\ ,-2D{\left\langle d_{\alpha }\right\rangle }^{\left(1\right)}d_{\alpha }-2D\left\langle d_{\alpha }\right\rangle d_{\alpha }^{\left(1\right)}+\frac{2}{N_{e}}D\left\langle d_{\alpha }\right\rangle {\left\langle d_{\alpha }\right\rangle }^{\left(1\right)}S+\frac{1}{N_{e}}D{\left\langle d_{\alpha }\right\rangle }^{2}S^{\left(1\right)}] \end{array}$$

Using the expressions

21 $$N_{e}=2Tr\left(DS\right)$$

22 $$\left\langle d_{\alpha }\right\rangle =2Tr\left(Dd_{\alpha }\right)$$

the terms containing the derivative of the dipole moment ⟨*d*_α_⟩ cancel and we obtain the expression

23 $$\begin{array}{ll} Tr\left(2Dh^{\left(1\right)}\right) & =Tr\left[2D{\left(h^{e}\right)}^{\left(1\right)}\right]\\ & +\sum_{\alpha }Tr[2Dd_{\alpha }S^{-1}d_{\alpha }^{\left(1\right)}-2D\left\langle d_{\alpha }\right\rangle d_{\alpha }^{\left(1\right)}\\ & +S^{-1}d_{\alpha }Dd_{\alpha }S^{-1}S^{\left(1\right)}+\frac{1}{N_{e}}D{\left\langle d_{\alpha }\right\rangle }^{2}S^{\left(1\right)}] \end{array}$$

That can be used to write the derivative contribution as

24 $$\begin{array}{l} G{\left(D\right)}_{\mu \nu }=G^{e}{\left(D\right)}_{\mu \nu }+\sum_{\alpha \rho \sigma }\left(2D_{\rho \sigma }{\left(d_{\alpha }\right)}_{\mu \nu }{\left(d_{\alpha }\right)}_{\rho \sigma }-D_{\rho \sigma }{\left(d_{\alpha }\right)}_{\mu \sigma }{\left(d_{\alpha }\right)}_{\rho \nu }\right)\\ ,=G^{e}{\left(D\right)}_{\mu \nu }+\sum_{\alpha }\left(2{\left(d_{\alpha }\right)}_{\mu \nu }\sum_{\rho \sigma }D_{\rho \sigma }{\left(d_{\alpha }\right)}_{\rho \sigma }-\sum_{\rho \sigma }{\left(d_{\alpha }\right)}_{\mu \rho }D_{\rho \sigma }{\left(d_{\alpha }\right)}_{\sigma \nu }\right)\\ ,=G^{e}{\left(D\right)}_{\mu \nu }+\sum_{\alpha }\left(2Tr\left(Dd_{\alpha }\right){\left(d_{\alpha }\right)}_{\mu \nu }-{\left(d_{\alpha }Dd_{\alpha }\right)}_{\mu \nu }\right) \end{array}$$

which we can use to expand the derivative as

25 $$Tr\left(DG^{\left(1\right)}\left(D\right)\right)=Tr\left(DG^{e\left(1\right)}\left(D\right)\right)+Tr\left(2\left\langle d_{\alpha }\right\rangle Dd_{\alpha }^{\left(1\right)}-2Dd_{\alpha }Dd_{\alpha }^{\left(1\right)}\right)$$

Inserting the Fock-matrix and nuclear repulsion terms we obtain the final expression for the molecular gradient

26 $$\begin{array}{ll} E^{\left(1\right)} & =2Tr\left(D{\left(h^{e}\right)}^{\left(1\right)}\right)+Tr\left(DG^{e\left(1\right)}\left(D\right)\right)\\ & -2Tr\left(DFDS^{\left(1\right)}\right)+h_{nuc}^{\left(1\right)}\\ & +2\sum_{\alpha }Tr\left(Dd_{\alpha }\left(S^{-1}-D\right)d_{\alpha }^{\left(1\right)}\right)\\ & -\sum_{\alpha }Tr\left(\left(S^{-1}d_{\alpha }Dd_{\alpha }S^{-1}-\frac{{\left\langle d_{\alpha }\right\rangle }^{2}}{N_{e}}D\right)S^{\left(1\right)}\right) \end{array}$$

This expression has been implemented in a development version of the eT program,^31^ where the first four terms are evaluated by calling the HF gradient routine with the modified Fock matrix, while the remaining terms are added as extra contributions. The most expensive part of evaluating the gradient is the second term in eq 26. To reduce computational cost, we used both the 4-fold symmetry of the two-electron integrals and screening. The details can be found in Horn et al.,^35^ but we have used the Cauchy-Schwarz (CS) inequality.^36^

## 4 Static External Fields

### 4.1 Electric Fields

The contribution of a static electric field to the molecular Hamiltonian is given by^20^

27 $$H_{ef}=-E_{ext}\cdot d$$

to the electronic Hamiltonian, where **d** is defined in eq 3, and ***E***_ext_ is the static electric field expressed in a.u. (one a.u. of electric field is E_h_/*ea*_0_ = 5.142 × 10^11^ V/m). The full Hamiltonian is expressed as

28 $$H=H_{qed}+H_{ef}+H_{\mathrm{int}}$$

The interaction term *H*_int_ vanishes due to the linearity of Maxwell’s equations, as the static electric field and the quantum electromagnetic field oscillate at different frequencies and therefore do not couple. The molecular gradients is obtained as in eqs 11 and 18 with modified one-electron integrals.

29 $$h=h^{e}-E_{ext}\cdot d$$

30 $$h^{\left(1\right)}={\left(h^{e}\right)}^{\left(1\right)}-E_{ext}\cdot d^{\left(1\right)}$$

### 4.2 Magnetic Fields

The contribution of a static magnetic field to the molecular Hamiltonian is given by^37^

31 $$H_{mf}=-\frac{1}{2}\sum_{pq}{\left(B_{ext}\cdot L_{0}\right)}_{pq}E_{pq}+\frac{1}{8}\sum_{pq}{\left(B_{ext}^{2}r_{0}^{2}\right)}_{pq}E_{pq}-\frac{1}{8}\sum_{pq}{\left(B_{ext}\cdot r_{0}\right)}_{pq}^{2}E_{pq}$$

where ***B***_ext_ is the external magnetic field expressed in a.u. (one a.u. of magnetic field is *B*_0_ = 2.35 × 10^5^ T).^37^ In eq 31 ***L***_0_ and ***r***_0_ are defined as

32 $${\left(L_{0}\right)}_{pq}=\langle p|\left(r-O\right)\times p)|q\rangle$$

33 $${\left(r_{0}\right)}_{pq}=\langle p|\left(r-O\right)|q\rangle$$

where ***r*** is the position operator, ***p*** is the momentum operator, and ***O*** is gauge origin. Combining this with the Pauli-Fierz Hamiltonian, we obtain the total Hamiltonian

34 $$H=H_{qed}+H_{mf}+H_{\mathrm{int}}$$

As shown earlier, the interaction term H_*int*_ vanishes due to the static nature of the magnetic field.^38^ The equations for the magnetic field molecular gradients have already been derived in the literature.^22,37^ The expressions for the energy and the gradient can be rewritten as in eqs 11 and 18, using a modified one-electron operator and its derivative

35 $$h=h^{e}-\frac{1}{2}B_{ext}\cdot L_{0}+\frac{1}{8}B_{ext}^{2}r_{0}^{2}-\frac{1}{8}{\left(B_{ext}\cdot r_{0}\right)}^{2}$$

36 $$h^{\left(1\right)}={\left(h^{e}\right)}^{\left(1\right)}-\frac{1}{2}B_{ext}\cdot L_{0}^{\left(1\right)}+\frac{1}{8}B_{ext}^{2}{\left(r_{0}^{2}\right)}^{\left(1\right)}-\frac{1}{8}{\left({\left(B_{ext}\cdot r_{0}\right)}^{2}\right)}^{\left(1\right)}$$

The main complication is evaluating the integrals, which should be done using London orbitals to ensure gauge-origin invariance of the energy.

## 5 Results and Discussion

As a first example, and also to validate our implementation, we study the effects of the three different fields on a single water molecule. We then apply our approach to simultaneous QED and electric fields. This is followed by an investigation of the inversion barrier in corannulene using QED and magnetic fields separately. Finally, we determined the equilibrium geometry of the water dimer in a QED field. All calculations were performed using uncontracted aug-cc-pVDZ (unc-aug-cc-pVDZ). We use an uncontracted basis to provide more flexibility, as the contraction coefficients in the standard basis sets are optimized without external fields.

### 5.1 Water Molecule

#### 5.1.1 Potential Energy Surface

We investigate the potential energy surface of the rotational degrees of freedom of a water molecule subjected to QED, electric, and magnetic fields. The equilibrium geometry of the water molecule was first optimized using the Hartree–Fock method. Subsequently, the potential energy surfaces were calculated by rotating a reference molecule chosen to be the equilibrium geometry in the HF around the *x*- and *y*-axes while maintaining the external fields along the positive *z*-axis.

The resulting potential energy surfaces for the three types of fields are shown in the Figure 1a–c. With their corresponding stationary points are labeled with Figure 1d–f.

**Figure 1:**
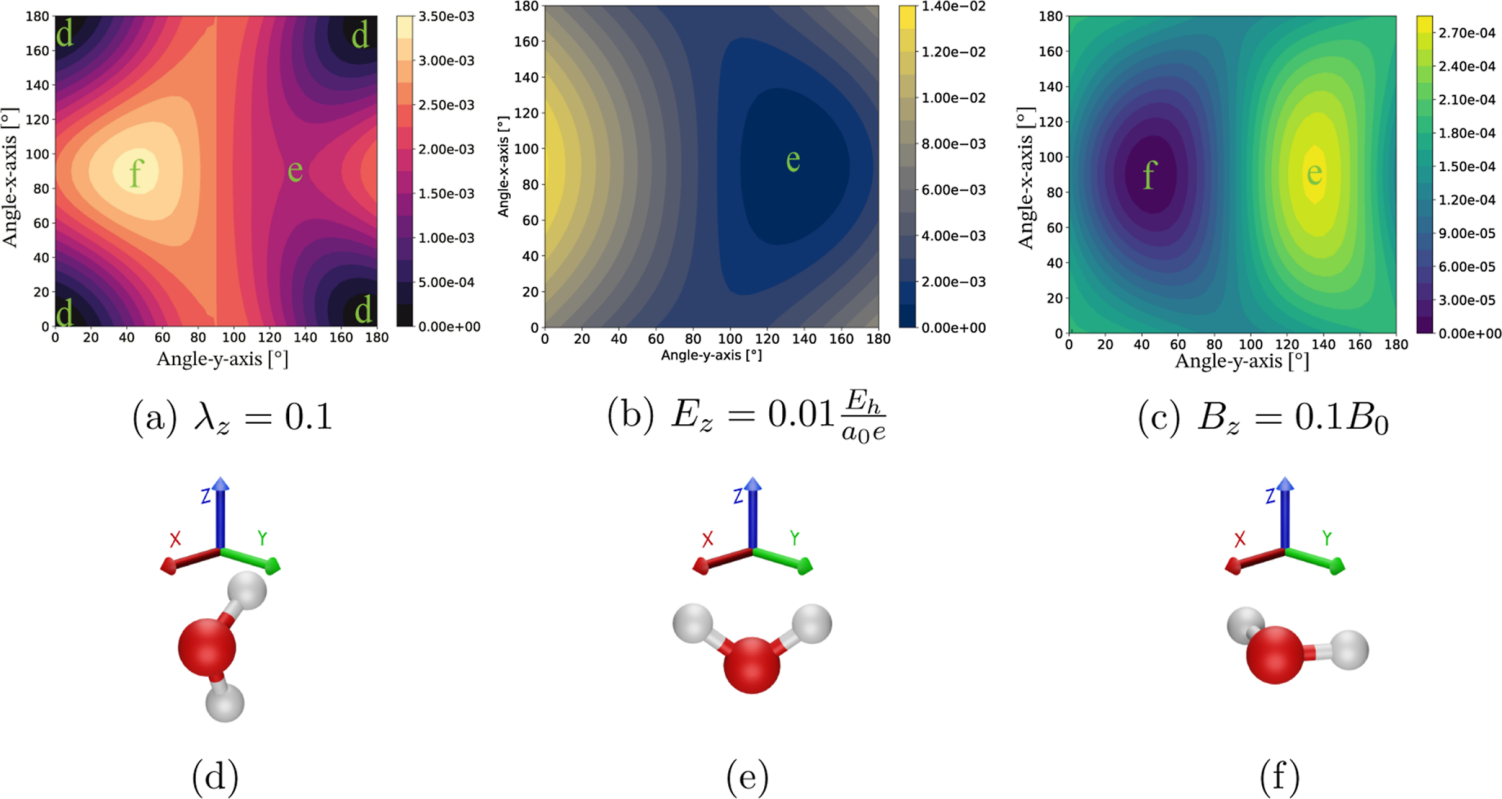
Figures a, b and c display, respectively, the potential energy surface of a water molecule in a QED field with λ_*z*_ = 0.1 au, an electric field with *E*_*z*_ = 0.01*E*_*h*_(*e* ^–1^ *a*_0_^–1^) and in a magnetic field with *B*_*z*_ = 0.1*B*_0_. The molecule is placed in the *xy* plane and rotated around the *x* (red) and *y* (green) axes with five-degree increments using an unc-aug-cc-pVDZ basis set with the field direction along the *z*-axis. The Figures d, e, and f depict the stationary points in their respective environments.

As shown in Figure 1, the electric field aligns the principal axis with the field direction The magnetic field orientation dependence of water has previously been described in the literature^22,37^ and is explained by the diamagnetic term in the magnetic Hamiltonian that resembles a harmonic oscillator potential in the directions orthogonal to the field. The effect of the QED field and the stationary points of the water can be explained by the photon field minimizing the fluctuation of the dipole along its polarization. This does not mean that the permanent dipole tends to be orthogonal to the field, but the QED field reduces the variance of the dipole. We note that the choice of a large λ is primarily intended to illustrate the effect. However, in practice, this choice of λ is not physically feasible due to the relationship between the size of the cavity and λ, given by $\lambda =\sqrt{4\pi /V}$ , where *V* is the volume of the cavity in a.u.^3,15^ To ease the comparison between the static electric field strength and the matter-light coupling, one can use the following identity for the electric field $E=\sqrt{\omega /2}\cdot \lambda$ in a.u. (in SI units $\lambda =\sqrt{\hbar /\left(\epsilon_{0}\cdot V\right)}$ ).

#### 5.1.2 Single Field Geometry Optimization

To verify the analytical gradient expressions obtained in Section 3, we performed a rigid geometry optimization and compared the result with the potential energy surfaces obtained in the previous subsection. The equilibrium geometries we obtained align with the findings of Figure 1. The geometry optimization procedure is based on a restricted step partitioned rational functional optimization (RS-P-RFO)^39^ and makes use of the translational rotational internal coordinate system (TRIC).^26,27^ Using the TRIC system permits us not only to include the orientational effects of the fields but also to investigate the changes in the internal degrees of freedom. Hence, we did a full geometry optimization and analyzed the bonds and angles with an increasing field strength. The results in Figure 2a,b, indicate that angle and bond length decrease with increasing coupling. In contrast, increasing the electric field strength results in an increased bond length but a decreased angle 2c, 2d. Finally, increasing the magnetic field strength leads to an increase in the angle and a reduction in the length of the bonds, as shown in Figures 2e,f.

**Figure 2:**
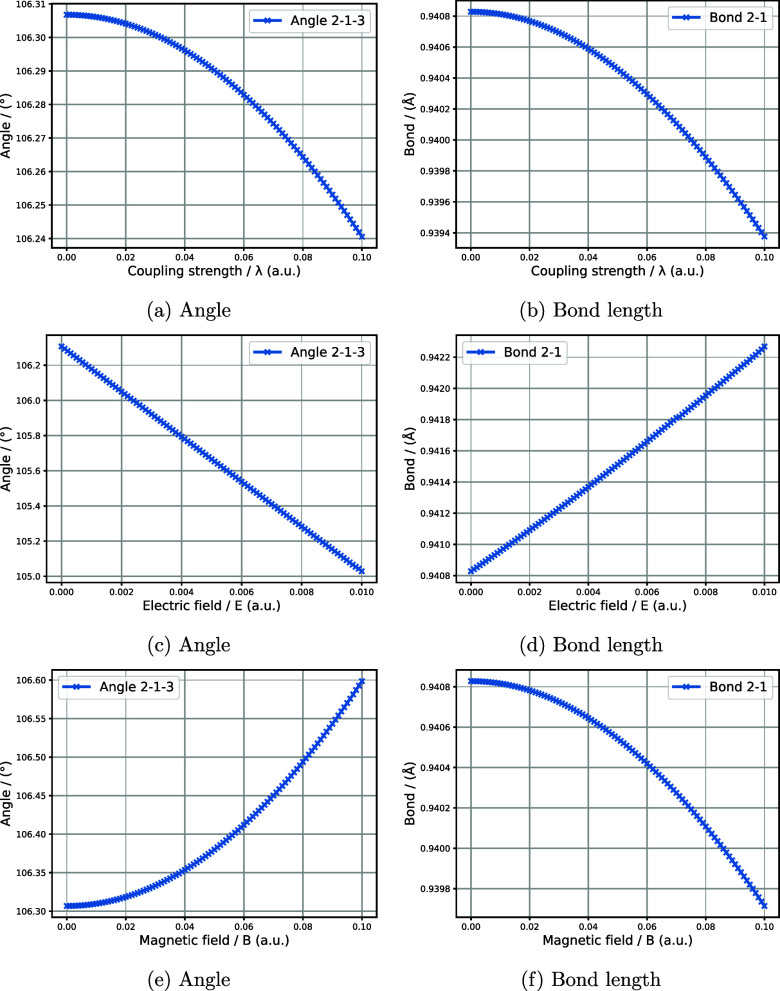
Figures a, c, e, and b, d, f display the changes in angles and bonds during a global geometry optimization for water when we increase the QED coupling, electric, magnetic field strengths. The geometry optimization was performed using an unc-aug-cc-pvtz basis set with the TRIC system.

#### 5.1.3 Dual Field Geometry Optimization

Building on the observed results from single-field interactions, we can rationalize the effects of dual-field configurations on water. When the fields are orthogonal, changing the field and coupling strengths tends to reduce angles, but the length of the bonds will depend on which fields are dominant, as observed in Figure 3a,b. Furthermore, the water molecule will have its preferred orientation with respect to both fields. However, having the fields parallel to each other, we can minimize the bond lengths with some combination of the fields, but the angles will be larger. Moreover, the molecules’ orientation to the fields will depend on the dominant effect.

**Figure 3:**
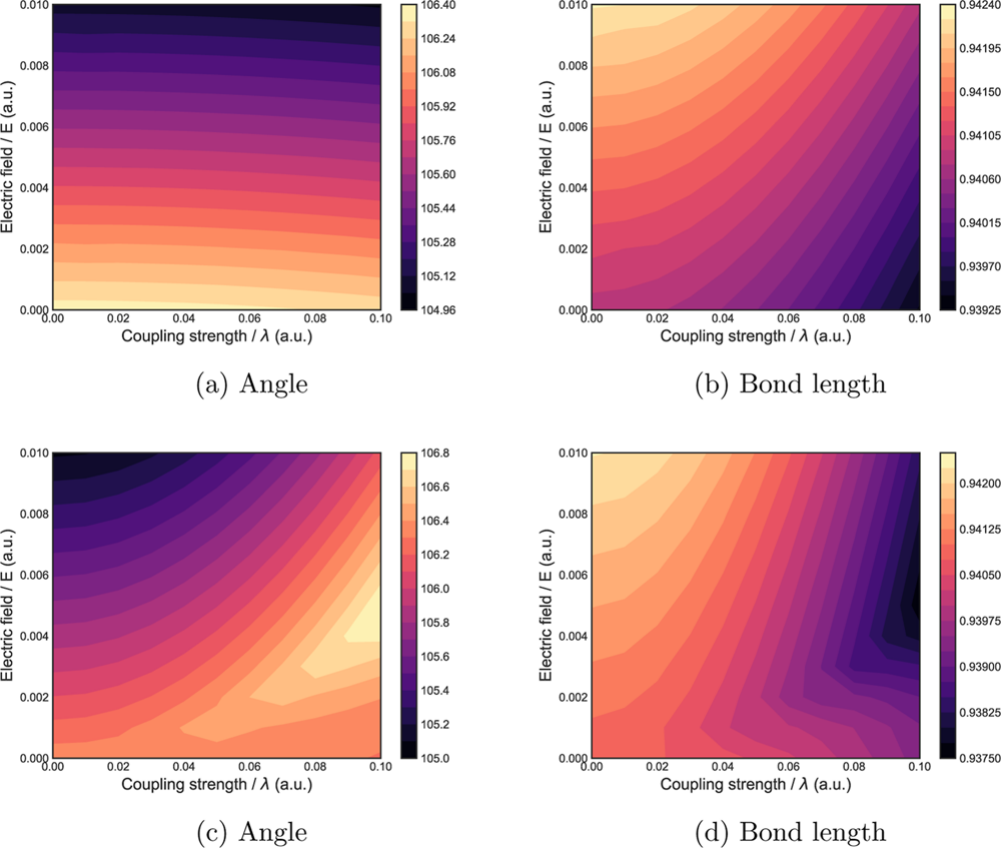
Figures a, b, c, d display the changes in angle and bond lengths during a global geometry optimization for water in a dual field when we increase the QED coupling λ and electric field strengths. The geometry optimization was performed using an unc-aug-cc-pvtz basis set with the TRIC system.

### 5.2 Corannulene

To investigate the effect on internal degrees of freedom in a larger system, we consider Corannulene (C_20_H_10_), a bowl-shaped molecule and investigate the inversion barrier^40^ under different external fields. We used magnetic field intensity from 0.0 *B*_0_ to 0.05 *B*_0_ with increments of 0.005 *B*_0_ and QED fields with couplings from 0.0 au to 0.05 a.u. with increments of 0.005 a.u. The inversion barrier in both magnetic and QED fields decreases as shown in Figure 4c,d. Hence, the equilibrium geometry becomes more planar with an increase in the strength of the fields. Regarding the orientation, in QED the principal axis of the molecule is aligned with the field, while in a magnetic field, it is orthogonal (as shown in Figure 4a,b). We did not include the results for the electric field because the double well configurations are not equivalent anymore. In particular, for a certain intensity of the electric field, the inversion barrier vanishes.

**Figure 4:**
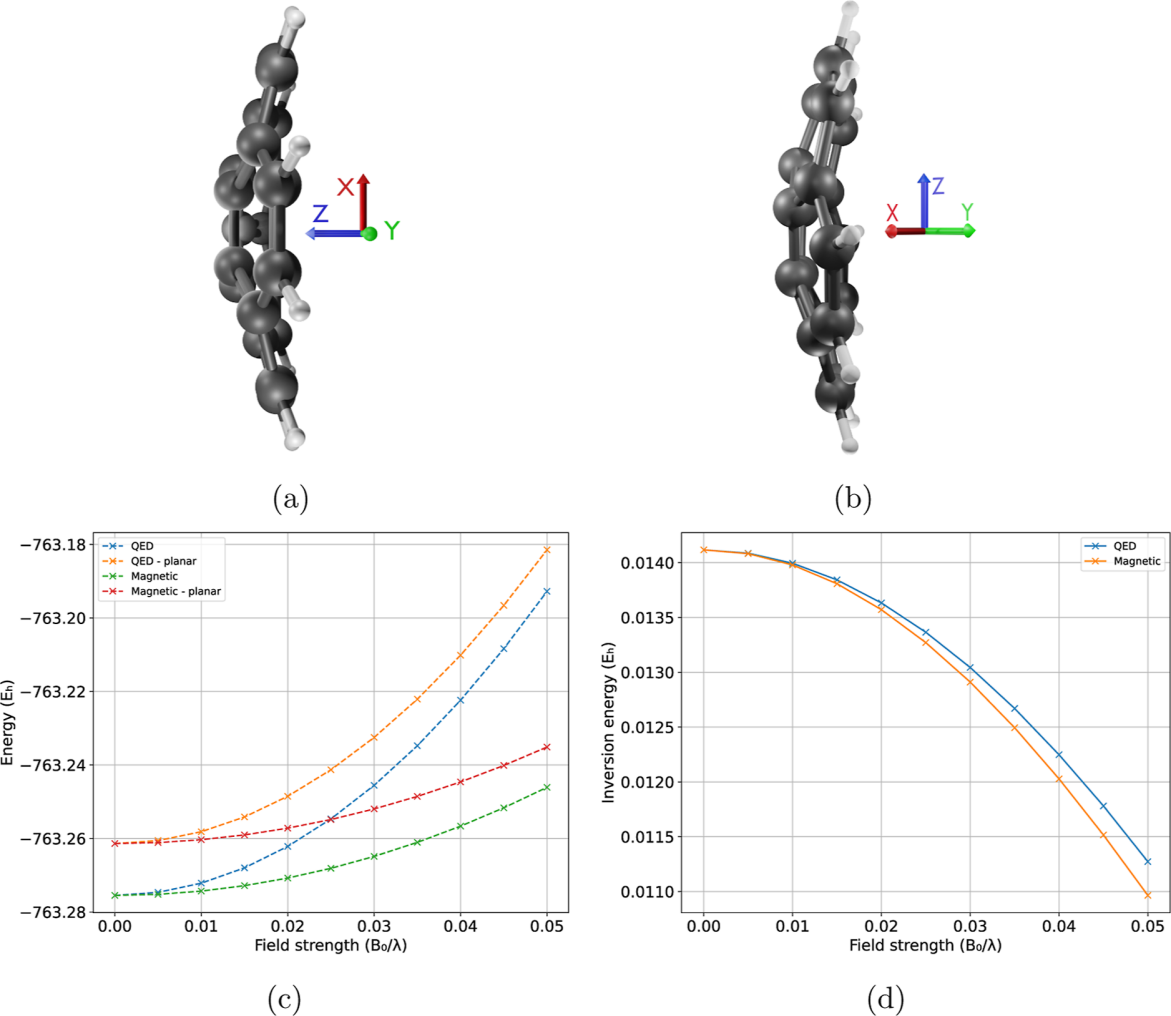
Figure a and b illustrate the alignment of corannulene relative to the external QED or magnetic fields, where *xyz* axes are red, green, and blue. Both the QED and magnetic fields are along *z* axis. Figure c shows the energies of the optimized geometries of corannulene in both its common and planar configurations as a function of increasing QED or magnetic field strengths. Lastly, Figure d depicts the inversion energy barrier under the same conditions.

### 5.3 Water Dimer

Our final application is to study the equilibrium geometry of the water dimer in a QED field. We performed a global and local optimization, increasing the coupling strength from 0.00 au to 0.1 au with 0.001 au increments, the results are displayed in Figure 5. We observed that globally, both water molecules have the hydrogen bond orthogonal to the polarization vector. In contrast, both water molecules prefer to be oriented locally, so their permanent dipoles are orthogonal to the field. This results in a shift of the dihedral angle from −120° to 0°, and a longer hydrogen bond (as displayed in Figure 5c,d).

**Figure 5:**
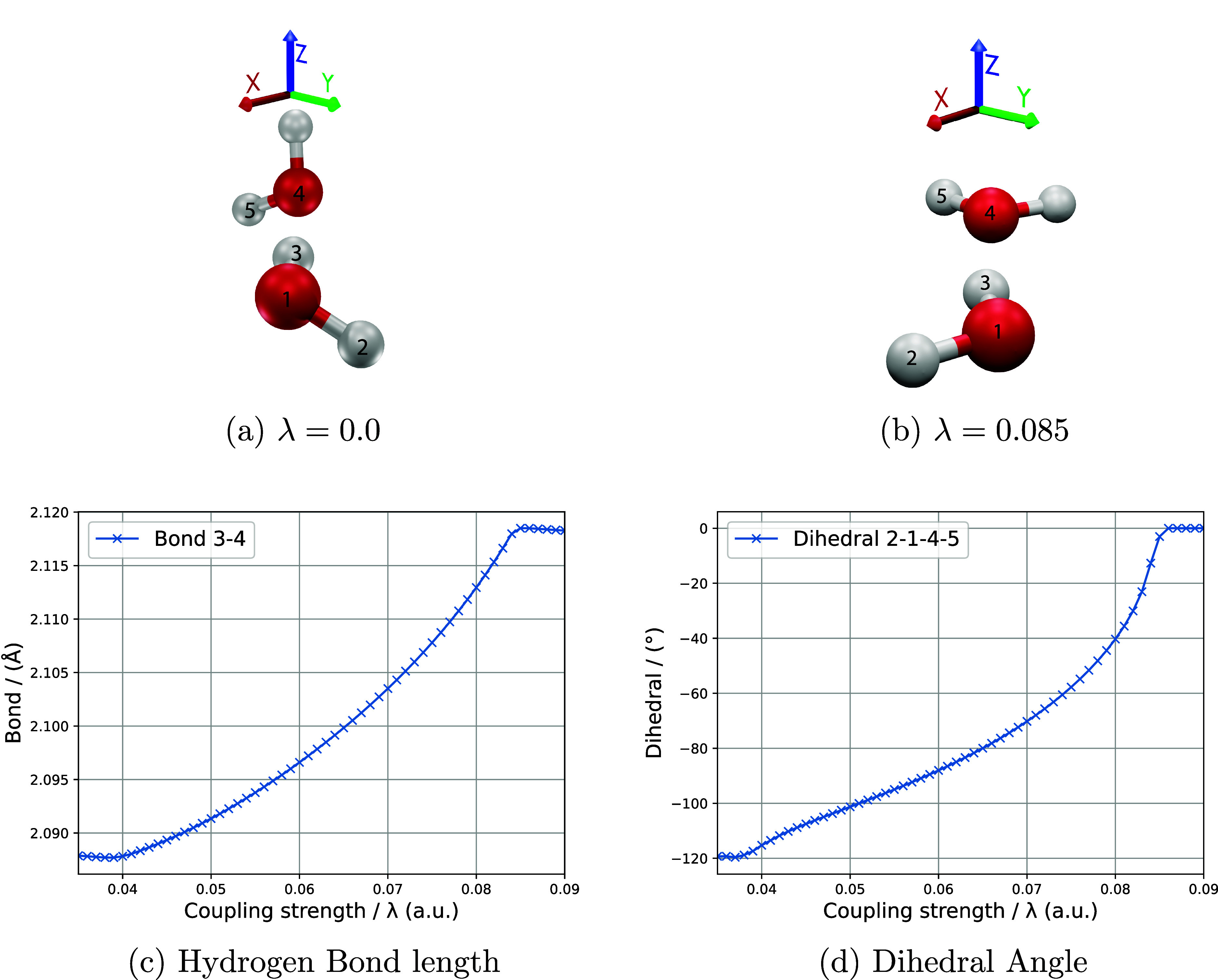
Figures show the equilibrium geometries of the water-dimer with a no field and b in a QED cavity with a coupling strength of λ = 0.085 au, and the polarization vector along the *z*-direction (blue). In c and d show the alterations in intermolecular parameters such as hydrogen bond length and dihedral angle, respectively, with increments of 0.01 au of the coupling strength λ.

Figure 6 depicts the change in the potential energy surface with an increasing coupling from 0.0 au to 0.1 au in increments of 0.01 au, keeping the dihedral angle fixed in the interval [-120°, 120°] with steps of 5°. The plot shows that the equilibrium geometry shifts from the HF configuration to the one with the dihedral angle of 0°. For the electric and magnetic fields applied in the previous section, the resulting changes in the dihedral angle are negligible, and observing any significant rotation requires field strengths several orders of magnitude higher.

**Figure 6:**
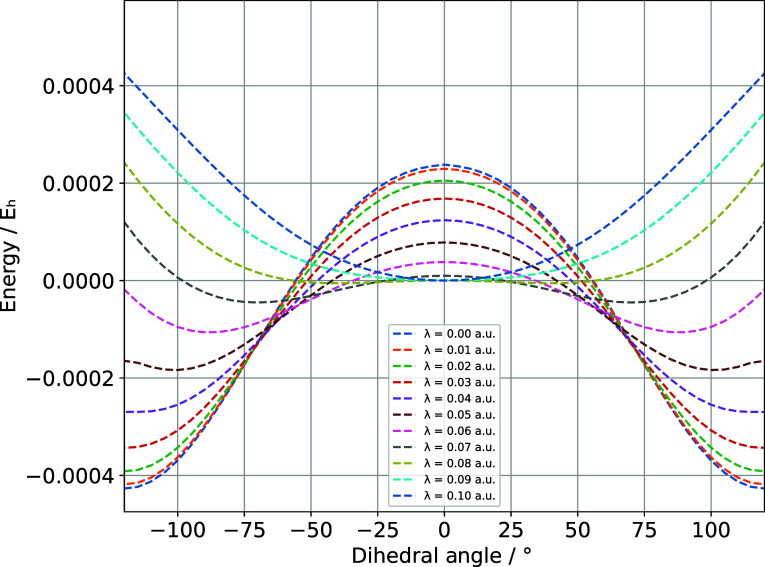
This plot was obtained performing a constrained geometric optimization of the water-dimer, fixing the dihedral angle in the range of −120° to 120° with increments of 5°, and increasing the coupling strength from 0.00 au to 0.10 au with increments of 0.01 au, using the unc-augment-cc-pvdz basis set.

## 6 Conclusions

In conclusion, we derived and implemented analytical molecular gradients for the QED-HF method and combined them with electric and magnetic fields. We applied the gradients to perform geometry optimization on water, water dimer, and corannulene. Single-field QED optimizations of water revealed a decrease in both bond lengths and angles with increasing light-matter coupling. The geometric changes in the molecule showed changes of 0.1 deg in the angles and 2 × 10^–3^ Å in bond lengths over the light-matter coupling range (0.0–0.1). These subtle geometric modifications are detectable through both rotational and vibrational spectroscopy, and vary under the influence of a matter-light coupling, as observed in recent theoretical studies.^41,42^ However, in real Fabry–Pérot cavity experiments, two field modes are present. Schnappinger and Kowalewski.^41^ suggest that using a single cavity mode may overestimate cavity effects. Under a static electric field, the structural perturbations increase by roughly a factor of 5, 0.5 deg, and 0.05 Å, which could be detectable spectroscopically.^43,44^ For magnetic fields we observe similar changes, but the required field strengths remain beyond current experimental capabilities.

We explored the combined effects of orthogonal and parallel dual fields composed of electric and QED fields. With orthogonal fields, the orientation of the water molecule was constrained, and the bond angle decreased, mirroring single-field behavior, while the behavior of the bond lengths depends on the dominant field component. The application of parallel fields enabled the selective minimization of bond lengths and maximization of the angle by tuning the electric field strengths and QED light-matter coupling. This observation contrasts with the behavior observed under single-field conditions, demonstrating the enhanced control offered by combining electric and QED fields.

We investigated the influence of these fields on the inversion barrier of corannulene. We observed a decrease in the inversion barrier with increasing field strength for both QED and magnetic fields, differing only in their orientation relative to the external field.

In the case of water-dimer, our results indicate that, with low coupling strengths, the dihedral angle between the two water molecules exhibits small changes. However, at sufficiently high coupling strengths, the global minima change, such that each water molecule will be locally oriented planar to the QED field.

Overall, these observations suggest that external fields, particularly in combination with QED fields, may influence molecular geometry in complex and system-dependent ways. While the trends identified offer interesting insights, further investigation is needed to fully understand the scope and consistency of these effects across different molecular systems.

## Data Availability

The Supporting Information is publicly available in ref 45.
